# Development of the Korean framework for senior-friendly hospitals: a Delphi study

**DOI:** 10.1186/s12913-017-2480-0

**Published:** 2017-08-04

**Authors:** Yoon-Sook Kim, Seol-Heui Han, Jeong-Hae Hwang, Jae-Min Park, Jongmin Lee, Jaekyung Choi, Yeonsil Moon, Hee Joung Kim, Grace Jung Eun Shin, Ji-Sun Lee, Ye Ji Choi, Kyeong Eun Uhm, In Ae Kim, Ji-Won Nam

**Affiliations:** 1 Department of Neurology, Konkuk University Medical Center, 120-1 Neungdong-ro (Hwayang-dong), Gwangjin-gu, Seoul, 05030 Korea; 2 Department of Health Administration, Hanyang Cyber University, 222 Wangsimri-ro, Seongdonggu, Seoul, 04763 Korea

**Keywords:** Senior friendly hospital, Accreditation, Elderly, Delphi survey

## Abstract

**Background:**

Aging is an inevitable part of life. One can maintain well-being and wellness even after discharge and/or transition if his or her functional decline is minimized, sudden decline is prevented, and functioning is promoted during hospitalization. Caring appropriately for elderly patients requires the systematic application of Senior-Friendly Hospital principles to all operating systems, including medical centres’ organization and environment, as well as patient treatment processes. The Senior-Friendly Hospital framework is valid and important for patient safety and quality improvement. This study aimed to make recommendations regarding the development of the Korean Framework for Senior-Friendly Hospitals for older patients’ care management, patient safety interventions, and health promotion, via a Delphi survey.

**Methods:**

Two rounds of Delphi surveying were conducted with 15 participants who had at least 3 years’ experience in accreditation surveying and medical accreditation standards, survey methods, and accreditation investigator education. In each round, we calculated statistics describing each standard’s validity and feasibility.

**Results:**

The Korean Framework for Senior-Friendly Hospitals included 4 Chapters, 11 categories, and 67 standards through consensus of the Senior-Friendly Hospitals task force and experts’ peer review. After the two rounds of Delphi surveying, validity evaluation led to no changes in standards of the Senior-Friendly Hospitals; however, the number of standards showing adequate validity decreased from 67 to 58. Regarding feasibility, no changes were necessary in the standards; however, the number of categories showing adequate feasibility decreased from 11 to 8 and from 67 to 30, respectively. The excluded categories were 3.2, 4.2, and 4.3 (service, transportation, and signage and identification). The highest feasibility values were given to standards 2.1.1, 4.1.4, and 4.1.6. The highest feasibility score was given to standard 2.4.2.

**Conclusions:**

The Korean Framework for Senior-Friendly Hospitals needs to include 4 Chapters, 8 categories, and 30 standards. The Accreditation Program for Healthcare Organizations should include Senior-Friendly Hospitals -relevant standards considering Korea’s medical environment.

## Background

The number of older persons aged 60 or more years will reach 1.2 billion by 2025, doubling the number in 2006. By 2050, this number will reach at least 2 billion and exceed the number of children under 15 [1]. In the Organisation for Economic Co-operation and Development (OECD), the proportion of older persons aged 60 or more years will be 14.3% in 2018 and exceed 20.8% in 2026, when the world will become super-aged [2]. With the increase of the older population in Korea, medical expenditures for their examination and treatment have exponentially increased and medical expenditures of older persons aged 65 or more have reached about 20 billion dollars annually [3].

Hospitals are optimized for younger adults who require quick diagnosis and medical or surgical treatment for a single disease; they are not ideal for frail elderly people with several comorbidities [4]. Elderly people often have difficulty adjusting to new environments; this may cause hospitalization to lead to functional decline, unexpected events, delayed treatment, and prolonged hospital days due to complications [4]. Accordingly, medical expertise alone often cannot restore elderly patients’ capacity for activities of daily living. Medical services for elderly people (e.g. Senior-Friendly Hospitals) should improve elderly people’s well-being, wellness, and well-dying by providing safety care systems and environments that optimize elderly people’s participation in health examination, particularly regarding respecting elderly people’s autonomy and self-led health care.

In 2004, the World Health Organization (WHO) published ‘Toward Age-friendly Primary Health Care’ , which recommended that health care professionals and administrators should regard age-friendly health care principles as a major reference when adjusting Primary Health Care (PHC) for an aging society [1]. The Age-friendly Principles address the following major areas: 1) Information, Education, Communication, and Training, including staff training in clinical geriatrics and approaches to patient education; 2) Health Care Management Systems; that is, adapting procedures (e.g. registration) to older persons’ specific needs and supporting continuity of care through updated medical records available at each visit; and 3) The Physical Environment; that is, clean and comfortable centres that maximally apply the principles of Universal Design [1]. Parke and Brand developed ‘The Elder-Friendly Hospital Initiative’ , a framework for elderly-friendly hospitals in Canada comprising four domains: 1) policies and procedures, 2) care systems, 3) social behavioural climate, and 4) physical design [5]. Taiwan has developed ‘Taiwan’s Framework of Age-friendly Hospitals’ , a framework for promoting healthy ageing in hospitals in Taiwan; this framework follows the WHO’s principles of age-friendly healthcare and Health Promotion Hospital (HPH) Standards. The framework consists of vision, values, mission, and strategies in four domains: 1) Management policy, 2) Communication and services, 3) Physical environment, and 4) Care processes [6]. The Ontario Senior-Friendly Hospitals framework offers evidence-based guidance for hospital-wide improvements in services for frail seniors within the following domains: 1) organizational support, 2) processes of care, 3) emotional and behavioural environment, 4) ethics in clinical care and research, and 5) physical environment [7, 8].

Though aging is an inevitable part of life, one can maintain well-being even after discharge and/or transition if functional decline is minimized, sudden decline is prevented, and functioning is promoted during hospitalization. Caring appropriately for elderly patients requires the systematic application of Senior-Friendly Hospitals principles to all operating systems, including medical centres’ organization and environment, as well as patient treatment processes.

This study aimed to make recommendations regarding the development of the Korean Framework for Senior-Friendly Hospitals for older patients’ care management, patient safety interventions, and health promotion, via a Delphi survey.

## Methods

### Deriving a Korean framework for senior-friendly hospitals

The Korean Framework for Senior-Friendly Hospitals was initiated by the Senior-Friendly Hospitals task force. The Senior-Friendly Hospitals task force was composed of clinical medicine (neurology, rehabilitation medicine, otorhinolaryngology-head and neck surgery, family medicine, respiratory and allergy medicine, and psychology), nursing, dietetics, pharmacy, gerontology, and quality improvement facilitation. The process is as follows.

First, Senior-Friendly Hospitals task force reviewed Taiwan’s Framework for Age-Friendly Hospitals [6] and Canada’s Senior-Friendly Hospitals self-assessment framework [9]. Second, standards were selected from the Taiwanese and Canadian frameworks, and then modified in consideration of the Korean situation. Third, a peer review was conducted by three geriatric experts. Finally, the Korean Framework for Senior-Friendly Hospitals was derived through consensus of the Senior-Friendly Hospitals task force and experts’ peer review. The Korean Framework for Senior-Friendly Hospitals consists of 4 Chapters, 11 categories, and 67 standards.

### Panel selection

We selected individuals with experience in developing and administering healthcare accreditation systems to examine the validity and feasibility of the Korean Framework for Senior-Friendly Hospitals. Eligible participants had experience in the development of accreditation standards and survey methods, development of educational programs for accreditation surveyors, and had been an accreditation surveyor for more than three years. Following previous research, Delphi methodology requires at least 10 expert participants [10]. Fifteen experts verbally agreed to participate.

### Research design

Delphi aims to make use of the positive attributes of interacting groups while removing the negative aspects largely attributed to the social difficulties within such groups. Accordingly, the following features characterize Delphi procedures: anonymity, iteration, controlled feedback, and statistical aggregation of responses.

Anonymity is achieved by collecting responses through questionnaires; this alleviates social pressure that might otherwise distort participants’ responses. Theoretically, anonymity allows participants to freely express their beliefs without feeling pressured by other participants and permits them to change their mind without loss of respect; this helps participants to consider ideas on merit alone.

Iteration is achieved by presenting questionnaires several times over a number of rounds, thereby allowing participants to change their opinion.

Controlled feedback takes place between rounds: each participant learns of the other participants’ opinions. Participants often receive this feedback as a simple statistical summary of responses (e.g. mean or median scores), although arguments may be presented. This ensures that all participants contribute to the discussion, not only the most vocal participants.

Statistical aggregation of responses is conducted at the procedure’s conclusion: the group’s judgment is expressed as a mean score and deviation of members’ opinions may be used to evaluate consensus. This provides richer information than a consensus decision [11].

The disadvantage of Delphi is that there are problems with the representation of experts, the reliability of anonymity, and the handling of extreme opinions. To complement the weaknesses of Delphi, the researchers continued to brainstorm regarding selecting Delphi experts and interpreting the results.

The researchers were blinded to the participants’ identity. We conducted two rounds of surveying to allow participants to revise their initial responses if they wished. In the second round of surveying, the mean, median, and mode, which were the result of the first round of survey were provided. The questionnaires that the participants completed during the first round were also sent by e-mail. In addition, the experts were able to express their opinions about the Senior-Friendly Hospitals in free text.

### Measures and analysis

The content validity ratio (CVR) [10] is a linear transformation of a proportional level of agreement. It represents the proportion of panel participants who rate an item as essential, and is calculated as follows:

$$ CVR=\frac{{\mathrm{n}}_{\mathrm{e}}-\left(\mathrm{N}/2\right)}{\mathrm{N}/2} $$

n_e_ is the number of panel members rating the item as essential and *N* is the number of panel members [12]. We had 15 panel members; therefore, the cut off value of CVR was 0.49. The CVR’s main benefit is to readily indicate if the level of agreement among panel members exceeds 50% [10, 12].

The Delphi participants responded to the first and second standards of Senior-Friendly Hospitals questions using 7-point Likert scales (1 = strongly disagree, 2 = disagree, 3 = slightly disagree, 4 = neutral, 5 = slightly agree, 6 = agree, and 7 = strongly agree). In previous research, 5 points or more were considered “acceptable” in 7-point Likert scales. We divided the scales into negative scores (1–3), neutral scores (4), and positive scores (5–7) [13, 14]. In each round, we calculated the mean, standard deviation, and CVR of each Senior-Friendly Hospitals standard’s validity and feasibility.

## Results

Participants’ demographics were as follows: 80% female, 86.7% currently working at a hospital, 13.3% working at Korea Institute of Healthcare Accreditation, average duration of experience in survey: 9.1 ± 3.2 years (range: 3–12 years; Table 1). All participants in the study responded to the two-round questionnaire.

**Table 1:** General characteristics (*N* = 15)

Variable	Category	n (%) or mean (SD)
Sex	Male	3 (20.0)
	Female	12 (80.0)
Affiliation	Hospital	13 (86.7)
	Korea Institute of Healthcare Accreditation	2 (13.3)
Duration of experience in surveying (years)		9.1 (3.2)

Regarding validity and feasibility, the first round of the Delphi survey showed that the CVR was below 0.49 (cut-off value); however, standards that showed a CVR over 0.49 in the second round are presented in Table 2.

**Table 2:** Delphi results

Chapters/Categories/Standards		Primary			Secondary		
		Mean	SD	CVR	Mean	SD	CVR
1. Management policy							
1.1 Developing a Senior-Friendly Hospitals							
1.1.1 The healthcare organization has policies and procedures for Senior-Friendly Hospitals.	Validity	6.4	1.0	0.87	6.4	0.9	0.87
	Feasibility	6.1	1.3	0.73	6.1	1.3	0.73
1.1.2 The healthcare organization develops a written age-friendly policy that values and promotes older persons’ health, dignity, and participation in care.	Validity	6.0	1.1	0.87	6.1	1.0	0.87
	Feasibility	5.9	1.3	0.73	5.8	1.2	0.73
1.1.3 The healthcare organization’s current quality and business plans identify age-friendliness as one of the priority issues.	Validity	5.7	1.2	0.87	5.7	1.0	0.87
	Feasibility	5.7	1.4	0.73	5.6	1.3	0.73
1.1.4 The healthcare organization has a department of administration for coordination and implementation of the age-friendly policy.	Validity	4.8	1.7	0.20	4.9	1.3	0.20
	Feasibility	3.9	1.8	−0.33	3.7	1.3	−0.60
1.2 Organizational support							
1.2.1 The healthcare organization identifies a budget for age-friendly services and materials.	Validity	5.9	0.9	0.87	5.9	0.6	1.00
	Feasibility	4.7	1.8	0.47	4.9	1.1	0.60
1.2.2 The healthcare organization improves the function of its information system to support implementation, coordination, and evaluation of the age-friendly policy.	Validity	5.9	0.8	1.00	6.0	0.7	1.00
	Feasibility	4.9	1.2	0.47	4.8	0.9	0.47
1.2.3 Qualified staff provides services to care for elderly people and their families.	Validity	5.7	1.4	0.60	5.9	1.1	0.73
	Feasibility	4.4	1.6	0.07	4.3	1.3	0.07
1.2.4 All staff receives basic training in age, gender, and culturally sensitive practices that address knowledge, attitudes, and skills.	Validity	5.7	1.0	0.87	5.8	0.7	1.00
	Feasibility	5.1	1.6	0.33	5.1	1.4	0.47
1.2.5 All clinical staff who provide care to older persons receive basic training in core competencies of elder care.	Validity	6.3	1.1	0.87	6.4	0.6	1.00
	Feasibility	5.9	1.5	0.60	5.9	1.4	0.60
1.2.6 The healthcare organization encourages best practices and innovations for senior-friendly service.	Validity	4.8	1.0	0.33	5.1	0.7	0.60
	Feasibility	4.2	1.3	−0.20	3.9	0.9	−0.47
1.2.7 Staff are involved in age-friendly policy-making, audit, and review.	Validity	4.8	1.6	0.47	5.3	0.8	0.73
	Feasibility	4.1	1.6	−0.20	3.9	1.0	−0.47
1.3 Continuous monitoring and improvement							
1.3.1 The healthcare organization has plans for quality improvement for Senior-Friendly Hospitals.	Validity	5.2	1.0	0.73	5.3	0.9	0.73
	Feasibility	5.1	1.4	0.47	4.9	1.0	0.60
1.3.2 A program for quality assessment of the age-friendly policy and its related activities is established. The assessment addresses development of organizational culture and perspectives of the seniors and the providers, as well as development of resources, performance of practices, and outcomes of care.	Validity	4.9	1.3	0.47	5.3	1.0	0.87
	Feasibility	4.7	1.5	−0.07	4.4	0.8	−0.33
1.3.3 The healthcare organization supports resources needed for quality improvement and patient safety activities.	Validity	6.2	1.1	0.87	6.4	0.6	1.00
	Feasibility	5.9	1.2	0.73	5.9	0.8	0.87
1.3.4 The healthcare organization includes sex- and age-specific analysis in its measurements of quality, safety, and patient satisfaction whenever appropriate. These data are available to staff for evaluation.	Validity	5.6	1.1	0.87	5.5	0.7	1.00
	Feasibility	5.6	1.3	0.60	5.7	0.9	0.87
1.3.5 The healthcare organization has processes to ensure a senior-friendly lens is applied to patient experience processes (e.g. patient/family engagement, senior-specific patient satisfaction processes).	Validity	5.3	1.2	0.60	5.3	0.8	0.73
	Feasibility	5.0	1.4	0.20	5.1	0.8	0.47
1.3.6 The healthcare organization manages elderly patients’ satisfaction.	Validity	5.3	1.2	0.60	5.3	0.8	0.73
	Feasibility	5.2	1.3	0.47	5.1	0.8	0.73
1.3.7 Results for patient satisfaction are reported to the leadership and shared with the associated staff.	Validity	5.2	1.3	0.60	5.4	0.7	0.87
	Feasibility	5.2	1.1	0.47	4.9	0.8	0.60
2. Care process							
2.1 Patient assessment							
2.1.1 The medical staff performs early initial assessment of patient’s needs for health promotion and disease prevention, including lifestyle, nutritional status, psycho-social-economic status, fall prevention, etc.	Validity	6.5	0.7	1.00	6.7	0.5	1.00
	Feasibility	5.8	1.0	0.87	5.6	0.8	0.87
2.1.2 The discharge plan is established and recorded according to the patient condition, at admission as inpatients.	Validity	6.0	1.1	0.73	6.0	0.8	0.87
	Feasibility	4.5	1.1	−0.07	4.5	0.5	−0.07
2.1.3 The healthcare organization has protocols for assessment of patient’s condition-related needs for health promotion, disease management, and rehabilitation.	Validity	6.1	0.8	1.00	6.0	0.5	1.00
	Feasibility	5.1	1.0	0.47	4.8	0.6	0.47
2.1.4 The assessment is documented in the patients’ record.	Validity	5.8	0.9	0.87	5.7	0.8	0.87
	Feasibility	5.4	1.2	0.60	5.2	0.9	0.60
2.1.5 The healthcare organization has guidelines on high-risk screening for seniors.	Validity	6.3	1.0	0.87	6.4	0.9	0.87
	Feasibility	5.0	1.3	0.20	4.9	0.6	0.47
2.1.6 Doctors for outpatient care carry out assessments for first-visit patients and complete the first-visit record (initial assessment record).	Validity	5.9	1.3	0.73	6.1	0.9	0.87
	Feasibility	4.7	1.6	−0.07	4.1	1.0	−0.60
2.1.7 Use of medications is reviewed at admission and regularly at outpatient services.	Validity	5.9	1.3	0.73	6.0	0.9	0.87
	Feasibility	4.4	1.4	0.07	4.5	0.7	0.20
2.1.8 The assessment of a patient’s needs is done at first contact with the healthcare organization and is kept under review and adjusted as necessary according to changes in the patient’s clinical condition or on request.	Validity	5.6	1.4	0.60	5.9	0.8	0.87
	Feasibility	4.5	1.6	−0.07	4.6	0.9	−0.07
2.2 Intervention and management							
2.2.1 Doctors provide the patients (and caregivers) information about patients’ health condition and involve them in the care process.	Validity	5.8	0.9	0.87	5.7	0.6	1.00
	Feasibility	4.9	1.2	0.20	4.8	0.9	0.33
2.2.2 Doctors re-establish care planning according to major changes in the patients’ conditions.	Validity	5.7	1.2	0.73	5.8	0.7	0.87
	Feasibility	5.0	1.4	0.33	4.8	0.9	0.60
2.2.3 The intervention and expected results are documented and evaluated in the records.	Validity	5.7	1.1	0.87	5.7	0.6	1.00
	Feasibility	5.2	1.4	0.60	5.0	1.0	0.73
2.2.4 Information on healthy aging and information on specific risks or conditions is available to patients, families, visitors, and staff.	Validity	5.9	0.9	0.87	5.8	0.9	0.73
	Feasibility	4.6	1.5	0.07	4.5	0.9	−0.07
2.2.5 Information given to the patient (and the caregiver) is recorded in the patient’s record.	Validity	5.7	1.0	0.73	5.7	0.7	1.00
	Feasibility	5.2	1.4	0.33	5.3	1.0	0.60
2.2.6 Clinical departments incorporate health promotion, rehabilitation, and risk management into their clinical practice guidelines or pathways as appropriate.	Validity	5.1	1.2	0.47	5.1	0.7	0.73
	Feasibility	4.4	1.1	−0.07	4.1	0.8	−0.33
2.2.7 Diagnostic investigations and procedures should accommodate age-related changes, tolerance, and ability.	Validity	6.2	0.9	1.00	6.1	0.7	1.00
	Feasibility	5.1	1.4	0.60	4.9	0.9	0.73
2.2.8 The healthcare organization provides multidisciplinary assessment, intervention, and evaluation of seniors.	Validity	6.1	0.9	1.00	5.9	0.7	0.87
	Feasibility	4.2	1.6	−0.07	4.0	1.1	−0.47
2.3 Community partnership and continuity of care							
2.3.1 A list of health and social care providers working in partnership with the healthcare organization is available.	Validity	4.7	1.3	0.20	4.9	0.9	0.33
	Feasibility	5.5	1.6	0.60	5.3	1.2	0.60
2.3.2 The healthcare organization provides to the patients (and caregivers) information about other health and social care providers.	Validity	4.9	1.5	0.33	5.1	0.8	0.47
	Feasibility	5.2	1.7	0.33	5.0	1.1	0.33
2.3.3 Qualified staff is in charge of referral services and the healthcare organization has an operation process.	Validity	4.7	1.4	0.07	4.5	1.2	0.07
	Feasibility	4.7	1.9	0.07	4.6	1.4	0.07
2.3.4 There is a written plan for collaboration with partners to improve the patients’ continuity of care.	Validity	4.5	1.6	0.07	4.7	1.4	0.33
	Feasibility	4.9	1.6	0.33	4.9	1.0	0.60
2.3.5 There is an agreed upon procedure for information exchange practices between organizations for all relevant patient information.	Validity	5.3	1.4	0.47	5.4	1.1	0.73
	Feasibility	5.1	1.7	0.33	5.1	1.2	0.60
2.3.6 Patients (and their families as appropriate) are given understandable follow-up instructions at out-patient consultation, referral, or discharge.	Validity	5.9	1.2	0.73	6.0	1.0	0.87
	Feasibility	5.1	1.0	0.60	5.0	0.8	0.73
2.3.7 The receiving organization is given in timely manner a written summary of the patient’s condition and health needs, and interventions provided by the referring organization.	Validity	5.7	1.2	0.73	5.9	1.0	0.87
	Feasibility	5.3	1.5	0.47	5.3	1.0	0.73
2.3.8 (Optional) A plan for rehabilitation describing the role of the organization and the cooperating partners is documented in the patient’s record.	Validity	5.1	1.4	0.33	5.1	0.9	0.60
	Feasibility	3.9	1.8	−0.20	3.5	1.1	−0.73
2.3.9 (Optional) The healthcare organization provides outreaching care services to the community elders.	Validity	4.1	1.4	−0.33	3.7	0.7	−0.87
	Feasibility	3.5	1.6	−0.47	3.2	0.9	−0.73
2.4. Ethical management of healthcare organization							
2.4.1 The healthcare organization has processes to solicit and follow patients’ advance directives that address care planning issues beyond “Do Not Resuscitate” orders.	Validity	6.3	1.0	0.87	6.5	0.8	0.87
	Feasibility	5.5	1.2	0.73	5.3	1.0	0.73
2.4.2 The healthcare organization has processes to deal with elder abuse issues when they are suspected or identified.	Validity	6.6	0.5	1.00	6.6	0.5	0.87
	Feasibility	6.0	1.0	0.87	6.2	0.8	1.00
3. Communication and services							
3.1 Communication							
3.1.1 The healthcare organization staff speaks to older persons in a respectful manner using understandable language and words.	Validity	6.0	1.1	0.87	6.1	0.6	1.00
	Feasibility	5.3	1.0	0.73	5.1	0.6	0.73
3.1.2 Information on the operation of the healthcare organization, such as opening hours, medical expenses, and registration procedures is provided in an age-appropriate way.	Validity	5.7	1.2	0.73	5.8	0.8	1.00
	Feasibility	5.1	1.1	0.47	4.9	0.6	0.47
3.1.3. Display the printed educational materials designed for the elderly; display pictures or materials.	Validity	5.9	1.0	0.87	5.9	0.7	1.00
	Feasibility	5.1	1.2	0.33	4.9	0.8	0.47
3.1.4 The healthcare organization provides adequate information and involves the older persons and their families at all stages of care.	Validity	6.4	0.6	1.00	6.3	0.6	1.00
	Feasibility	5.4	1.1	0.73	5.3	0.8	0.73
3.1.5 The healthcare organization respects older persons’ ability and right to make decisions on their care.	Validity	6.0	0.8	1.00	6.1	0.6	1.00
	Feasibility	4.9	1.1	0.20	4.7	0.7	0.07
3.2 Service							
3.2.1 The healthcare organization makes every effort to adapt its administrative procedures to the special needs of older persons, including older persons with low educational levels or with cognitive impairments.	Validity	6.0	1.0	0.87	6.1	0.6	1.00
	Feasibility	4.7	1.6	0.07	4.7	1.0	0.20
3.2.2 The healthcare organization identifies and supports older persons with financial difficulties to receive appropriate care.	Validity	5.9	0.9	0.87	5.9	0.6	1.00
	Feasibility	4.8	1.1	0.33	4.9	0.7	0.47
3.2. The healthcare organization has volunteer programs to support patients and visitors in reception, navigation, transport, reading, writing, accompanying, or other help as appropriate in outpatient and inpatient services.	Validity	5.4	0.8	0.87	5.2	0.8	0.73
	Feasibility	4.9	1.3	0.20	4.8	0.9	0.33
3.2.4 The healthcare organization has a volunteer program that provides opportunities for older persons, including community seniors, patients, and their families to participate in the healthcare organization’s volunteer services.	Validity	4.7	1.3	0.33	4.8	1.0	0.47
	Feasibility	4.7	1.5	0.07	4.6	0.8	0.07
4. Physical environment							
4.1 General environment and equipment							
4.1.1 The healthcare organization has a policy for facilities that is considered a senior-friendly view (Universal protocol, CODE-plus, etc.).	Validity	6.0	0.8	1.00	5.8	0.7	1.00
	Feasibility	4.8	1.3	0.20	4.7	1.0	0.20
4.1.2 The facilities, including waiting areas, are clean and comfortable throughout.	Validity	5.5	1.2	0.73	5.2	0.7	0.87
	Feasibility	4.7	1.8	0.20	4.6	1.2	0.33
4.1.3 The facilities are equipped with good lighting, non-slip floor surfaces, stable furniture, and clear walkways.	Validity	6.1	0.8	1.00	6.1	0.6	1.00
	Feasibility	5.3	1.5	0.33	5.3	1.1	0.73
4.1.4 The toilet and bathing facilities and heads of the healthcare organization beds are equipped with an emergency alarm system.	Validity	6.5	0.8	1.00	6.7	0.7	1.00
	Feasibility	6.1	1.3	0.73	6.1	1.2	0.87
4.1.5 The healthcare organization has barrier-free washrooms equipped with basic washing facilities.	Validity	6.4	0.8	1.00	6.6	0.7	1.00
	Feasibility	5.3	1.5	0.47	5.5	1.1	0.87
4.1.6 There are hand railings on both sides of hallways.	Validity	6.5	0.8	1.00	6.7	0.7	1.00
	Feasibility	5.9	1.3	0.73	6.0	1.1	0.87
4.1.7 Bed heights are appropriate for older persons.	Validity	6.4	0.8	1.00	6.5	0.7	1.00
	Feasibility	5.0	1.6	0.47	5.1	1.2	0.73
4.2 Transportation							
4.2.1 The main healthcare organization premises has convenient transportation connections.	Validity	4.9	1.7	0.33	4.9	1.0	0.47
	Feasibility	3.5	1.5	−0.73	3.4	0.8	−1.00
4.2.2 The healthcare organization’s main entrance has a passenger drop-off/pick-up area and there is staff providing assistance.	Validity	4.9	1.2	0.47	5.0	0.7	0.87
	Feasibility	4.1	1.2	−0.20	3.9	0.9	−0.47
4.2.3 For people with disabilities, there is enough space for them to get on/off and they are provided with mobility aids (e.g. wheelchair).	Validity	5.7	1.0	0.87	5.3	0.6	0.87
	Feasibility	5.0	1.6	0.33	4.9	0.7	0.33
4.3 Signage and identification							
4.3.1 Simple and easily readable signage is posted throughout the healthcare organization to facilitate orientation and personalize providers and services.	Validity	5.3	1.3	0.33	5.4	1.1	0.60
	Feasibility	5.1	1.5	0.20	5.1	1.3	0.33
4.3.2 The healthcare organization applies common signage for direction and makes it easy for older persons to identify.	Validity	5.3	1.3	0.33	5.4	1.1	0.60
	Feasibility	4.9	1.4	0.20	4.8	1.3	0.20
4.3.3 Healthcare staff is easily identifiable using name cards.	Validity	5.1	1.4	0.33	5.1	1.1	0.47
	Feasibility	5.1	1.5	0.20	4.9	1.2	0.20

*CVR* content validity ratio

The following standards indicated adequate validity (CVR 0.49 or more): 1.2.6 (The healthcare organization encourages best practices and innovations for senior-friendly service.), 1.2.7 (Staff are involved in age-friendly policy-making, audit and review.), 1.3.2 (A program for quality assessment of the age-friendly policy and its related activities is established. The assessment addresses development of organizational culture and perspectives of the seniors and the providers, as well as development of resources, performance of practices and outcome of care.), 2.2.6 (Clinical departments incorporate health promotion, rehabilitation, and risk management into their clinical practice guidelines or pathways as appropriate.), 2.3.5 (There is an agreed upon procedure for information exchange practices between organizations for all relevant patient information.), 2.3.8 (A plan for rehabilitation describing the role of the organization and the cooperating partners is documented in the patient’s record.), 4.2.2 (The healthcare organization’s main entrance has a passenger drop-off/pick-up area and there is staff providing assistance.), 4.3.1 (Simple and easily readable signage is posted throughout the healthcare organization to facilitate orientation and personalize providers and services.), and 4.3.2 (The healthcare organization applies common signage for direction and makes it easy for older persons to identify; see Table 2).

The following standards indicated adequate feasibility (CVR 0.49 or more): 1.2.1 (The healthcare organization identifies a budget for age-friendly services and materials.), 1.3.1 (The healthcare organization has plans for quality improvement for Senior-Friendly Hospitals.), 1.3.7 (Results on patient satisfaction are reported to the leadership and shared with the associated staff.), 2.2.2 (Doctors re-establish care planning according to major changes in the patients’ conditions.), 2.2.5 (Information given to the patient (and the caregiver) is recorded in the patient’s record.), 2.3.5 (There is an agreed upon procedure for information exchange practices between organizations for all relevant patient information.), 2.3.7 (The receiving organization is given in a timely manner a written summary of the patient’s condition and health needs, and interventions provided by the referring organization.), 4.1.3 (The facilities are equipped with good lighting, non-slip floor surfaces, stable furniture, and clear walkways.), 4.1.5 (The healthcare organization has barrier-free washrooms equipped with basic washing facilities.), and 4.1.7 (Bed heights are appropriate for older persons.; see Table 2).

The following are additional comments from Delphi participants. 1) It is very important to introduce Senior-Friendly Hospitals in Korea. Government support is needed to introduce new frameworks for Senior-Friendly Hospitals and apply them to hospitals. 2) It is necessary to make a change in the medical environment for the vulnerable elderly. It seems that a framework for Senior-Friendly Hospitals can be a new paradigm for elderly care in Korea. 3) Rather than developing a framework for Senior-Friendly Hospitals separately, it is necessary to develop an integrated accreditation program that is included in Korea’s accreditation program for healthcare organizations.

## Discussion

Older persons have unique needs: chronic conditions and co-morbidity, different manifestations, high utilization of healthcare, and vulnerability to hospitalization and healthcare. In this study, older persons said they suffered from unfriendliness of healthcare [6]. Frontline healthcare staff is not familiar with common elderly problems (e.g. falls, incontinence, immobility, and confusion). Sometimes, health and medical problems are not perceived as urgent by patients if they have unresolved family or social problems [1]. Therefore, it is necessary to establish a Senior-Friendly Hospitals system in Korea for elderly patient-centred care.

The Senior-Friendly Hospitals accreditation standards contained 4 Chapters, 11 categories, and 67 standards. After the two rounds of Delphi surveying, validity evaluation led to no changes in the standards of Senior-Friendly Hospitals; however, the number of standards showing adequate validity decreased from 67 to 58. Regarding feasibility, no changes were necessary in the standards; however, the number of categories showing adequate feasibility decreased from 11 to 8 and from 67 to 30, respectively. The excluded categories were 3.2, 4.2, and 4.3 (service, transportation, and signage and identification); these categories were lower than the cut-off value of CVR (0.49) for all standards. An Age-Friendly Hospitals framework in Taiwan includes four domains: management policy, care processes, communication and services, and physical environment [6]. An organization-wide framework for Elder Friendly Hospitals in British Columbia, Canada uses four domains: policies and procedures, care systems, social behavioural climate, and physical design [5]. An age-friendly hospital in Quebec, Canada consists of four domains: integrated process of geriatric care across the organization, transitions management across acute care and the community, clinical decision-making assistance, and optimized physical environments [8]. Canada has a senior-friendly hospital domain similar to Taiwan. However, if you look at the contents of each domain, Canada did not emphasize service and transportation similar to Korea.

The highest feasibility values were given to standards 2.1.1 (The medical staff performs early initial assessment of patient’s needs for health promotion and disease prevention, including lifestyles, nutritional status, psycho-social-economic status, fall prevention, etc.), 4.1.4 (The toilet and bathing facilities and heads of the healthcare organization beds are equipped with an emergency alarm system.), and 4.1.6 (There are hand railings on both sides of hallways.: mean, 6.5; CVR, 1.00). The highest feasibility score was given to standard 2.4.2 (The healthcare organization has processes to deal with elder abuse issues when they are suspected or identified; mean, 6.2; CVR, 1.00). Iran reported it would provide timely healthcare services for senior-friendly hospitals. In order to do so, special screening for the elderly is performed at admission to hospital [15]. Taiwan and Canada also recognized the need for patient care processes and adequate physical environments, and included them in the framework of senior-friendly hospitals [6, 9]. The Accreditation Program for Healthcare Organizations in Korea is designed to encourage medical institutions to continuously and voluntarily work towards enhancing patient safety and care quality, thereby providing quality medical services to the public [16]. Since adopting the Accreditation Program for Healthcare Organizations in 2010, accredited hospitals prioritize patient safety and care quality improvement. Accordingly, all participants in this research supported the Program’s validity, resulting in a CVR of 1. Additionally, all participants supported the feasibility of items pertaining to elder abuse, resulting in a CVR of 1.

Senior-Friendly Hospitals respects elderly patients’ autonomy and encourages self-care through safe care systems and environments, thereby improving elderly people’s well-being, wellness, and well-dying [17]. The Senior-Friendly Hospitals frameworks developed in Taiwan [6] and Canada [9] aim to provide safe and high-quality medical services to frail elderly people with various comorbidities. Accordingly, this research aimed to use Delphi methodology to develop a framework for Senior-Friendly Hospitals that is adapted to Korea’s specific situation. Finally, the cut-off value of the CVR was 58 for validity and 30 for feasibility among the 60 standards.

Korea’s Accreditation Program for Healthcare Organizations assesses hospitals holistically and is internationally certified. In addition, numerous evaluations are imposed by institution-recommended requirements, medical insurance fees, and related laws; for instance, the Emergency Medical Service Institutions Evaluation, Mental Institutions Assessment, Stroke Unit Certification, Endoscopy Unit Accreditation, and the Healthcare Provider for International Patients Certificate.

Each hospital must complete at least four accreditations and assessments. Accordingly, the framework for Senior-Friendly Hospitals is valid and important regarding patient safety and quality improvement. However, Delphi experts working in hospitals seem to be burdened by the accreditation program for elderly patients. This may be why the framework for Senior-Friendly Hospitals that only targeted elderly people received low feasibility scores. Accreditation systems in Korea should include survey items specifically targeting elderly people. Therefore, the Accreditation Program for Healthcare Organizations should include Senior-Friendly Hospitals -relevant standards (Table 3).

**Table 3:** Relationship between healthcare organization accreditation standards and feasible standard of senior-friendly hospitals

Healthcare organization accreditation standard	Feasible standard of senior-friendly hospitals
2.1 [Required] There is a management system for quality improvement and patient safety at the organization level.	1.3.1 The healthcare organization has plans for quality improvement for Senior-Friendly Hospitals.
	1.3.3 The healthcare organization supports resources needed for quality improvement and patient safety activities.
2.4 The healthcare organization plans and implements indicator management at the organization level.	1.3.4 The healthcare organization includes sex- and age-specific analysis in its measurements of quality, safety, and patient satisfaction whenever appropriate. These data are available to staff for evaluation.
	1.3.6 The healthcare organization manages elderly patients’ satisfaction.
	1.3.7 Results of the reported patient satisfaction is reported to the leadership and shared with the associated staff.
3.2.2 The healthcare organization identifies the health care needs of inpatients, and carries out initial assessments or reassessments of the patients.	2.1.1 The medical staff performs early initial assessment of patient’s needs for health promotion and disease prevention, including lifestyle, nutritional status, psycho-social-economic status, fall prevention, etc.
	2.1.4 The assessment is documented in the patients’ record.
3.1.5 The healthcare organization provides discharge, transfer, and referral services to maintain care continuity.	2.3.1 A list of health and social care providers working in partnership with the healthcare organization is available.
	2.3.4 There is a written plan for collaboration with partners to improve the patients’ continuity of care.
	2.3.5 There is an agreed upon procedure for information exchange practices between organizations for all relevant patient information.
	2.3.6 Patients (and their families as appropriate) are given understandable follow-up instructions at out-patient consultation, referral, or discharge.
	2.3.7 The receiving organization is given in a timely manner a written summary of the patient’s condition and health needs, and interventions provided by the referring organization.
4.1.1 The healthcare organization establishes and carries out care plans and goals in a timely manner to maintain appropriate patient care.	2.2.2 Doctors re-establish care planning according to major changes in the patients’ conditions.
	2.2.3 The intervention and expected results are documented and evaluated in the records.
	2.2.5 Information given to the patient (and caregiver) is recorded in the patient’s record.
	2.2.7 Diagnostic investigations and procedures should accommodate age-related changes, tolerance, and ability.
	3.1.4 The healthcare organization provides adequate information and involves the older persons and their families at all stages of care.
4.2.2 The healthcare organization provides high-quality medical care to patients who require cardiopulmonary resuscitation.	2.4.1 The healthcare organization has processes to solicit and follow patients’ advance directives that address care planning issues beyond “Do Not Resuscitate” orders.
7.1.2 The healthcare organization protects the rights and safety of vulnerable patients.	1.1.1 The healthcare organization has policies and procedures for Senior-Friendly Hospitals.
	1.1.2 The healthcare organization develops a written age-friendly policy that values and promotes older persons’ health, dignity, and participation in care.
	2.4.2 The healthcare organization has processes to deal with elder abuse issues when they are suspected or identified.
	3.1.1 The healthcare organization staff speaks to older persons in a respectful manner using understandable language and words.
8.1 The leadership carries out reasonable decision-making and operates the healthcare organization under a systematic plan.	1.1.3 The healthcare organization’s current quality and business plans identify age-friendliness as one of the priority issues.
	1.2.1 The healthcare organization identifies a budget for age-friendly services and materials.
9.2 The healthcare organization provides continuous education and training to the staff.	1.2.5 All clinical staff who provide care to older persons receive basic training in core competences of elder care.
11.1 The healthcare organization carries out safety management of the facility and environment.	4.1.3 The facilities are equipped with good lighting, non-slip floor surfaces, stable furniture, and clear walkways.
	4.1.4 The toilet and bathing facilities and heads of the healthcare organization beds are equipped with an emergency alarm system.
	4.1.5 The healthcare organization has barrier-free washrooms equipped with basic washing facilities.
	4.1.6 There are hand railings on both sides of hallways.
	4.1.7 Bed heights are appropriate for older persons.

## Conclusion

In this study, a Korean framework for Senior-Friendly Hospitals was reconstructed with reference to Taiwan’s Framework of Age-friendly Hospitals [6] and Canada’s Senior-Friendly Hospitals self-assessment framework [9]. This study suggests that the Ministry of Health and Welfare and the Korea Institute for Healthcare Accreditation should consider the Korean framework for Senior-Friendly Hospitals to develop an integrated accreditation system by including the 58 valid and 30 feasible standards in the Accreditation Program for Healthcare Organizations.

### Limitations

The term ‘Senior-Friendly Hospitals’ is fairly unfamiliar to Korean people. Even though an explanation of Senior-Friendly Hospitals was provided to the Delphi participants when sending the questionnaire, additional explanations were often requested during the survey. As the concept of Senior-Friendly Hospitals is unfamiliar to the participants, this might have influenced how people answered.

## Abbreviations

CP: Clinical pathway
CPG: Clinical practice guidelines
CVR: Content validity ratio
HPH: Health promotion hospital
OECD: Organisation for economic co-operation and development
PHC: Primary health care
WHO: World Health Organization
